# Honesty, candour, and transparency: clinical implications

**DOI:** 10.1080/17571472.2016.1146464

**Published:** 2016-03-10

**Authors:** Andrew Papanikitas

**Affiliations:** ^a^ Philosophy and Ethics Editor, LJPC, London, UK

**Keywords:** Ethics, duty, candour

## Abstract

In advance of a medical conference on the duty of candour for medical ethics educators, this paper discusses the duty of candour as a significant development in the culture of medicine. Those who teach medical ethics need to assess its implications for their own practice. It is also a duty that needs to be critically examined in light of both patients’ interests and clinical work environments if it is to be practical and not meaningless rhetoric. Two examples of ways in which that critical examination might take place are outlined.

## Why this matters to me

The duty of candour is one which is expected of all healthcare workers. It is not an easy duty to fulfil. Education needs to equip healthcare workers with ethical coping strategies for realities of practice as well as providing them with worthy ideals.

## Key messages

Those who teach medical ethics need to assess the implications of the duty of candour in practice, and healthcare workers need realistic ways of putting candour into practice.

The Institute of Medical Ethics is running their tenth medical education conference on Friday 11 March 2016 in London on the theme of: ‘Honesty, candour and transparency: clinical implications’. The aim of the conference is to explore the clinical implications of the professional duty of candour, which applies when things go wrong with a patient’s care, and the GMC’s more general requirement to be honest and open with patients or carers when discussing care, treatment and prognosis.

The Mid-Staffordshire scandal and the Montgomery case challenge clinicians to be candid in their dealings with patients. All UK healthcare workers must at all times be honest with their patients but this can be manifested in a variety of ways that fit the situation of each patient without interfering with the clinician’s duty of care. This is a significant development in the culture of medicine and those who teach medical ethics need to assess its implications for their own practice. It is also a duty that needs to be critically examined in light of both patients’ interests and clinical work environments if it is to be practical and not meaningless rhetoric. Below, I outline two examples of ways in which that critical examination might take place.

For example, although the consensus is that deceiving patients is generally wrong, Sokol [1] suggests that benignly intended deception may be morally acceptable in certain circumstances. He proposed that a deception flowchart might help clinicians make better informed decisions regarding deception and might be useful when teaching medical students and clinicians. Sokol’s flowchart has two key safety checks to ensure the permissibility of the proposed deception. The first is for the doctor to consider how he or she would articulate and defend their views and reasoning before a body of reasonable people, such as a professional association or a court of law. The purpose of this test is to encourage doctors to reassess the strengths and weaknesses of their justifications, to reduce the risk of personal bias and self-deception in their judgments and to remind them that deceiving patients is no trivial matter. The examples used by Sokol are extreme and potentially debatable though the flowchart itself appears intuitive.

While honesty, in general, is in the best interests of patients, doctors may find these duties challenging to apply in practice. They may be concerned about the impact on their careers of complying with the duty of candour and raising concerns about patient safety.

Posting on her online blog, www.clearer-thinking-co.uk, Shale outlines the issue for care-givers [2]:

Professional caregivers often experience the organisational response to harm as threatening, ostracising, and unsupportive…

We all know that a blame culture is dangerous because it inhibits organisational learning. We now need to acknowledge that it is also cruel and unjust. It increases stress on professionals, demoralises and demotivates them, and contributes to the deterioration in clinical performance that sometimes follows unexpected outcomes.

When a patient suffers significant harm, caregivers are likely to experience a strong psychological reaction. In the immediate aftermath, they have to deal with their own response and also with disclosing the event to the patient. As time goes on, they find themselves having to contend with unfamiliar and often bewildering organisational processes, and experiencing the reactions of everyone around them with an interest in the case. This could include the patients’ supporters, professional colleagues, managers, regulators, insurers, lawyers, and perhaps the coroner or police.

To expect professionals to cope with all this without thoughtful support is both morally wrong and frankly dangerous.

How, therefore, do we prepare medical students and other junior healthcare staff effectively for the challenges which they will face, not only in the future but also now, as students, who may witness poor practice or errors that affect patient safety? Integrity or being true to robust moral beliefs and professional norms can be severely tested when institutions are less than supportive.

Speakers at the IME conference will explore legal and ethical aspects of the duties of candour and honesty, and the complexities encountered in actual clinical practice, drawing from experience in different specialties including general practice, obstetrics and anaesthesia. There will be the opportunity to discuss personal ideas and experience with a focus on guiding the development and delivery of teaching on these issues. The conference will be of interest to anyone involved in teaching medical students and other clinicians, whether in an academic or clinical setting. Medical students themselves are welcome at IME conferences. Details about the meeting can be found on the Institute of Medical Ethics Website (itself a resource for educational meetings and resources)[3].
